# Routing Protocols for Wireless Body Area Networks: Recent Advances and Open Challenges

**DOI:** 10.3390/s26010231

**Published:** 2025-12-30

**Authors:** Haoran Qin, Haoru Su, Xiaopeng Niu, Hongli Chen

**Affiliations:** 1 College of Computer Science, Beijing University of Technology, Beijing 100124, China; qinhaoran@emails.bjut.edu.cn (H.Q.); chenhongli@bjut.edu.cn (H.C.); 2 School of Computer and Artificial Intelligence, Beijing Technology and Business University, Beijing 100048, China; nxp_btbu@btbu.edu.cn

**Keywords:** wireless body area network, routing protocol, Internet of Things, quality of service, energy efficiency

## Abstract

The growing demand for personalized healthcare is driving the development of Wireless Body Area Networks (WBANs). These networks enable continuous monitoring of physiological parameters. In WBANs, routing protocols are essential for ensuring reliable data delivery. However, designing efficient protocols is challenging due to the specific environment of the human body. Key issues include limited energy, frequent topology changes caused by movement, and diverse Quality of Service needs. In this review, we investigate, summarize, and analyze state-of-the-art WBAN routing protocols. Specifically, we outline the architecture of WBAN-based eHealth systems and review major design challenges. We then present a categorized survey of recent protocols. Subsequently, we examine the distribution across protocol categories and compare their performance. Finally, we identify open challenges and discuss future research directions.

## 1. Introduction

The global trend of aging populations and a rising demand for personalized healthcare have established smart healthcare and the Internet of Things as active research areas. Within this context, Wireless Sensor Networks (WSNs) are widely studied and used in many scenarios. A specialized branch of WSNs, Wireless Body Area Networks (WBANs), has emerged with a specific focus on human health monitoring. WBANs enable continuous, remote monitoring of physiological data through miniature sensors implanted in or employed on or near the human body. Architecturally, a WBAN’s communication protocol stack is often guided by standards like IEEE 802.15.6, with the network layer being responsible for data forwarding from sensor nodes to a central coordinator.

The routing protocol is an essential component of this network layer technology. Its primary function is to determine data transmission path selection and resource allocation, making its performance critical to the overall performance of the network. There are three main network topologies used in WBANs: star, tree, and mesh, with the latter two being multi-hop topologies. In these networks, the core routing challenge is to efficiently and reliably transmit data from various sensor nodes to the data coordinator, ensuring that both routine and critical health data are delivered according to their specific requirements.

However, the specific human-centric architecture of WBANs introduces a series of multiple interrelated design challenges that routing protocols must overcome. These challenges, which distinguish WBANs from traditional WSNs, include: extremely limited energy resources for nodes, making energy efficiency a primary concern; a highly dynamic network topology due to human movement, which can cause frequent link disruptions; varying Quality of Service (QoS) requirements for different medical data types; and potential biosafety concerns related to the radio frequency radiation absorbed by human tissue. Overviews and comparisons of WBAN routing protocols are provided in [1,2].

Given these challenges, the development of routing protocols capable of addressing them has attracted substantial research attention in the WBAN field. Several survey articles have summarized the recent state of development in the field. However, the rapid evolution of the field necessitates an updated review to cover recent developments. This paper complements the existing literature by providing a survey of recent advancements in the field. Our discussion is based on reading and summarizing representative studies on WBAN routing, and organizing their main ideas and design trade-offs. To structure this review, we formulated the following three primary Research Questions (RQs) at the outset of our study: RQ1: What is the current taxonomy of state-of-the-art WBAN routing protocols published between 2020 and 2025 based on their main design concerns and technical paradigms (e.g., QoS, temperature, posture/movement, cross-layer/SDN control, and learning-based decision-making)? RQ2: How do different categories of protocols compare in terms of critical performance metrics such as energy efficiency, latency, and reliability? RQ3: What are the core unresolved challenges and future research directions for designing next-generation WBAN routing protocols? The main contributions are summarized as follows: First, it reviews WBAN routing protocols published between 2020 and 2025, organizing them into nine categories to reflect common design concerns and implementation paradigms in recent work. Second, it presents a comparative analysis of these categories, highlighting their respective performance characteristics and limitations. Third, it identifies unresolved challenges and outlines potential directions for future research to support the advancement of WBAN technology.

To identify recent studies on WBAN routing, we performed a targeted literature search in IEEE Xplore, Web of Science, ACM Digital Library, and MDPI. We used combinations of keywords such as “wireless body area network”, “WBAN”, “routing”, “energy efficiency”, “QoS”, “thermal-aware”, and “mobility”. We then screened titles/abstracts and read the full text of papers that are clearly related to routing at the intra-WBAN layer. Our goal is to summarize representative approaches and organize them into an interpretable taxonomy, rather than to exhaustively include all publications or to perform a meta-analysis.

The remainder of this paper is organized as follows: Section 2 introduces the fundamental architecture of WBANs. Section 3 details the unique routing challenges inherent in WBANs. Section 4 presents the recently proposed WBAN routing protocols in a categorized manner. Section 5 compares the protocol categories at a high level, summarizes commonly reported performance aspects and trade-offs, and discusses practical considerations such as deployment feasibility and security. Section 6 discusses the unresolved challenges in the field and explores promising future research directions. Finally, Section 7 concludes the paper.

## 2. WBAN-Based eHealth System Architecture

**Figure 1:**
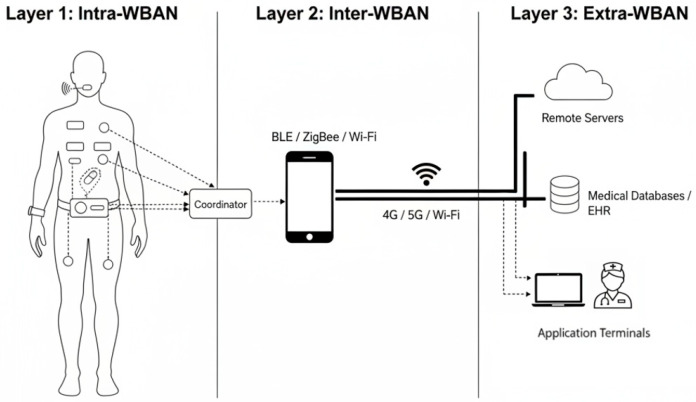
Typical three-layer communication architecture of a WBAN-based eHealth system.

To understand the design complexity of WBAN routing protocols, it is necessary to examine them within the comprehensive framework of a WBAN-based eHealth system. In this integrated ecosystem, the WBAN serves as the vital sensing front-end. Its primary task is to capture sensitive physiological information and ensure it is successfully transmitted to remote medical centers for diagnosis and monitoring. This enables a shift from periodic, episodic health checks to continuous, real-time patient monitoring. The reliability of this initial data hop is foundational; any failure at this on-body stage compromises the entire diagnostic chain. As illustrated in Figure 1, this eHealth architecture is logically divided into three distinct layers to manage this process efficiently. This layered approach allows for a separation of concerns, where each layer can be independently optimized for its specific function. This hierarchical organization defines the complete path of data transmission, starting from the initial acquisition on the human body and ending with processing on backend servers. Each transition between these layers involves a different communication technology and presents unique routing challenges. This multi-layered structure sets the specific operational environment and constraints for the routing protocol. For instance, the limited processing power of on-body nodes and the variable connectivity to gateway devices directly shape the design requirements for any feasible routing algorithm. An effective protocol must therefore be layer-aware, optimizing its behavior based on its specific position within this end-to-end data pipeline. Therefore, this section provides a detailed breakdown of this foundational architecture and outlines the specific functions and responsibilities assigned to each layer.

### 2.1. Layer 1: Intra-WBAN

This is the foundational layer of the WBAN-based eHealth system architecture, directly interacting with the user’s body. This layer consists of a series of miniaturized biosensor nodes and a central coordinator.

- Sensor Nodes: These nodes serve as the primary sources for data acquisition and can be categorized into two types: wearable devices, such as ECG electrode patches applied to the skin or wrist-worn blood pressure monitors; and implantable devices, such as pacemakers or glucose monitors placed inside the body. They share the common characteristics of being extremely small in size and consuming minimal power, responsible for converting collected physiological signals (such as electrical signals, pressure, temperature, etc.) into digital data.
- Coordinator: The coordinator is typically a slightly more powerful device, such as a dedicated data aggregator or a smartphone-integrated module. It serves as the central hub of the Intra-WBAN, responsible for managing sensor nodes within the network, collecting the data they transmit, and performing initial processing and aggregation. Data from all sensor nodes are first aggregated to the coordinator via single-hop or multi-hop communication.

### 2.2. Layer 2: Inter-WBAN

This layer is responsible for securely transmitting data collected from the human body to external networks. This layer is typically handled by the user’s personal smart device (Personal Device, PD), such as a smartphone, tablet, or dedicated gateway device. The PD connects to the coordinator at Layer 1 via short-range wireless technologies like Bluetooth Low Energy (BLE), ZigBee, or Wi-Fi to receive aggregated physiological data. Subsequently, it leverages its more robust communication capabilities—such as cellular networks (4G/5G) or Wi-Fi—to forward the data to remote servers at Layer 3.

### 2.3. Layer 3: Extra-WBAN

This layer is typically the destination and application layer for data, forming the backend support system for the entire smart healthcare service. This layer includes:

- Remote Servers: Deployed in cloud-based or healthcare institution data centers, these servers are responsible for receiving, storing, and processing vast amounts of physiological data.
- Medical Databases: These are used for the long-term storage of users’ electronic health records (EHR).
- Application Terminals: Authorized healthcare professionals, family members, or the user themselves can access analysis results via computers, mobile apps, and other terminals to obtain health reports, receive early warning notifications, and conduct remote diagnosis and intervention. In emergencies, the system may trigger alerts depending on the application design to notify emergency response centers.

## 3. Routing Challenges in WBANs

The specific architecture and human-centric nature of WBANs, as described in the previous section, necessitate that its routing protocol design strike a delicate balance among multiple interdependent challenges. These challenges are the primary drivers behind the diverse routing strategies discussed later in this paper.

### 3.1. Energy Efficiency

The miniaturization of sensor nodes is a prerequisite for the practical implementation of WBAN, but it also imposes extremely stringent resource constraints. Node battery capacity, processor speed, and memory size are all severely limited. For implanted nodes, battery replacement typically requires surgical intervention, which is costly and painful for users. Consequently, energy efficiency is often a key consideration in designing WBAN routing protocols. Every data transmission, reception, exchange of control information, and even idle channel monitoring during routing consumes precious energy. An efficient routing protocol must minimize communication overhead to extend the overall network’s lifespan.

### 3.2. Mobility and Topology Changes

Unlike traditional WSNs deployed in static environments, the carrier of WBANs is the active human body. Daily movements such as walking, running, turning, or bending cause frequent changes in the relative positions and distances between sensor nodes. This phenomenon is termed topological dynamism. It can cause previously reliable communication link quality to deteriorate sharply or even break completely, known as postural partitioning. This places extremely high demands on routing protocols: they must rapidly detect link failures and swiftly find alternative paths; otherwise, massive packet loss and transmission delays will occur.

### 3.3. QoS Assurance

Physiological data transmitted via WBAN exhibit diverse QoS requirements. For instance, routine monitoring data such as heart rate or body temperature demand minimal real-time performance but require reliable transmission. Conversely, alert data generated during critical events like arrhythmia or epileptic seizures should be delivered with low latency and high reliability. Routing protocols must possess the capability to distinguish between different data types and provide differentiated services. This includes establishing a logical “green channel” for priority transmission of critical packets. In practice, this is typically implemented through multi-priority queuing mechanisms, where emergency data bypasses standard buffers for preemptive scheduling, or via MAC-layer differentiation techniques—such as assigning Guaranteed Time Slots (GTS) or reduced backoff intervals—to ensure immediate medium access. This can help reduce the delivery time of alerts while maintaining routine traffic.

### 3.4. Overheating and Biosafety

Human tissue exhibits strong absorption and attenuation effects on electromagnetic waves, resulting in an extremely complex wireless channel environment for WBANs with path loss significantly higher than in free space. Additionally, heat generated by sensor nodes (especially implantable nodes) during operation is absorbed by surrounding body tissues through radiofrequency radiation. The Specific Absorption Rate serves as a key metric for evaluating this effect. If a routing protocol frequently schedules a node for high-power data forwarding, it may cause localized temperature increases at that node. Prolonged overheating can inflict irreversible damage on sensitive human tissues. Therefore, routing protocol design must not only account for channel attenuation but also incorporate thermal sensing mechanisms to prevent the formation of hotspots within the network.

### 3.5. Heterogeneous Traffic

Data flows within WBAN networks are highly heterogeneous. Different sensor types generate data at vastly different rates, packet sizes, and levels of criticality. For instance, an ECG sensor may produce a continuous data stream at hundreds of samples per second, while a temperature sensor might transmit only one data point per minute. Routing protocols must efficiently manage these heterogeneous data streams, preventing network congestion caused by high-bitrate streams while ensuring low-rate but potentially equally critical data are not starved due to channel contention.

## 4. Recent Routing Protocols for WBANs

Before delving into specific protocols, it is essential to define their scope within the architecture presented in Section 2. The routing protocols reviewed in this section primarily operate within Layer 1 (Intra-WBAN), responsible for data forwarding from sensor nodes to the coordinator. While Layer 2 and Layer 3 are vital for end-to-end connectivity in eHealth systems, the stringent constraints detailed in Section 3—such as extremely limited energy, tissue heating effects, and highly dynamic topology—are often most apparent at the Intra-WBAN level. As a result, many studies focus on optimizing communication within this layer to address these unique challenges. In this section, WBAN routing protocols are categorized based on their design principles and then examined in terms of their primary optimization objectives.

### 4.1. Cluster and Tree Routing Protocol

To achieve hierarchical network management, cluster and tree routing protocols organize sensor nodes into groups called clusters. This layered topology is often used in WBAN studies to organize multi-hop forwarding and reduce per-node transmission overhead. Within each cluster, a cluster head (CH) is selected, whose primary responsibility is to gather data from member nodes and transmit the aggregated information to the base station. The key to the significant energy efficiency of this two-tiered communication architecture is that it limits the vast majority of node communications to short-range, low-power links with the local CH. The energy-demanding long-range transmission task is often handled by the CH, thereby conserving the energy of member nodes. Consequently, the central design challenge for such protocols is the efficient selection of the optimal CH. This challenge requires the protocol to dynamically identify the most suitable node to act as CH, considering not only the minimization of total communication energy but also the candidate node’s own residual power. Periodic role rotation is essential to balance the network’s load and prevent network partitioning due to premature node failure. The recent research trend involves incorporating intelligent computational methods, especially hybrid swarm intelligence algorithms, to enhance CH election efficiency and network energy balance. For example, the MGWOQL [3] protocol merges Q-learning with a Modified Grey Wolf Optimization, using machine learning to predict energy consumption and the Q-learning-enhanced GWO algorithm to select the best CH. More recent studies are exploring even more complex hybrid models, such as the ABC-CSO [4] protocol, which uses the artificial bee colony (ABC) algorithm for CH election and the chicken swarm optimization (CSO) algorithm for data routing, balancing network energy consumption through this two-step process. This separation of duties allows each algorithm to focus on its strengths, creating a more robust overall solution. Similarly, the ABBSOA-MSSOA [5] protocol integrates the adaptive binary bird swarm optimization algorithm (ABBSOA) for cluster division with the modified snake swarm optimization algorithm (MSSOA) for path selection, both aiming to maximize the network’s operational lifetime. Table 1 summarizes these representative protocols, outlining the key challenges each one addresses and its limitations.

### 4.2. QoS-Aware Routing Protocol

QoS-aware routing protocols aim to provide differentiated QoS for heterogeneous medical data by prioritizing traffic. For instance, it selects routes with minimum delay and sufficient bandwidth for critical emergency data, often at the cost of higher energy consumption for that specific transmission. In a clinical context, such a trade-off is justified, as the imperative of ensuring patient safety in critical situations far outweighs the incremental energy expenditure. For the highest-priority emergency data, the PCRP [6] protocol typically employs the most direct and fastest transmission method. Protocols such as EQRD [7] and IM-QRP [8] focus on minimizing end-to-end delay for latency-sensitive data by optimizing bandwidth utilization and link reliability. Elaborating on this principle of prioritization, the Tripe-EEC [9] protocol by Ullah et al. proposes a more granular classification scheme, dividing data into four distinct classes: normal, on-demand, and two types of emergency data based on low and high thresholds. This detailed classification allows for more sophisticated, priority-based path allocation and conflict resolution, while also considering node temperature to avoid hotspots. This marks a step toward multi-metric QoS consideration. Recent research has begun integrating advanced multi-criteria decision-making (MCDM) methods. The ARAP [10] protocol employs MCDM and TOPSIS techniques to select relay nodes. This approach evaluates multiple conflicting QoS metrics, including node remaining energy, traffic load, and node importance, to select the globally optimal next-hop node, significantly enhancing network lifetime and energy efficiency. Table 2 summarizes these representative protocols.

### 4.3. Temperature-Sensing Routing Protocol

To ensure biosafety, temperature-sensing routing protocols treat node temperature as a core decision-making variable, proactively steering clear of transmission paths that could lead to localized overheating of human tissue. Initial research in this field was primarily focused on hotspot avoidance strategies. The ATAR [11] protocol uses a multi-ring routing technique to reactively find an alternative path when a node’s temperature rises, thereby distributing the traffic load to prevent tissue damage. Subsequent research evolved to consider temperature in conjunction with other key network performance metrics. The EOCC-TARA [12] protocol, designed by Ahmed et al., is an example; operating under a Software-Defined Network (SDN) architecture, it uses an Enhanced Multi-objective Spider Monkey Optimization (EMSMO) algorithm to co-optimize temperature, energy efficiency, and network congestion, representing a move toward more comprehensive multi-objective solutions. Building on this, the latest research has advanced to more sophisticated collaborative optimization techniques that are deeply integrated with cutting-edge intelligent algorithms. The SETA [13] protocol utilizes an improved ant colony optimization (ACO) algorithm to jointly consider multiple dimensions, such as temperature, energy efficiency, and link quality, to find globally optimal cooling routes. Under the SDN framework, the ETC-MOTLBO [14] protocol implements centralized, collaborative control over temperature, energy consumption, and congestion through a teaching-learning-based optimization (TLBO) algorithm. Furthermore, some protocols focus on acquiring more granular temperature data, such as the TSA-ESRP [15] protocol, which classifies node temperatures into several discrete levels and dynamically adjusts routing choices based on the node’s real-time thermal state. The increasing reliance on SDN frameworks and intelligent algorithms underscores a shift towards centralized, predictive, and holistic network management. In summary, these studies clearly outline the developmental trajectory of thermal management technology in WBANs: from initial simple, reactive hotspot avoidance to the recent complex, multi-objective, and intelligent thermal-aware routing strategies. Table 3 summarizes these representative protocols.

### 4.4. Posture/Movement-Sensing Routing Protocol

Posture/movement-sensing routing protocols address link disruptions due to human movement. Research has progressed from reactive strategies that repair links after disruption to more proactive approaches. One proactive mechanism involves predicting link stability. EMA-RPL [16] continuously monitors the received signal strength indicator (RSSI), using a weakening signal as a trigger to proactively switch routes before a link fails. RSSI-based prediction can be unreliable under multipath fading. Although not a WBAN routing study, the work in [17] shows that delay–Doppler representations can provide richer channel features than scalar RSSI by capturing time–frequency dispersion. Such features may offer a possible direction for future movement-aware routing, for example, by supporting more informative link or motion estimation. However, extracting and processing these features may require additional signal processing capability and measurement overhead. Whether this approach is practical for WBAN devices still needs validation. The ARMR [18] protocol integrates reinforcement learning (RL) with link lifetime estimation (LLE) to intelligently learn and select more durable routes. Another approach directly leverages the user’s physical context. The mechanism in MHRP [19] involves pre-deploying redundant nodes and activating only the specific set of nodes that offer the most stable communication path for the user’s recent posture (e.g., sitting vs. walking). Taking this context-aware approach further, in [20], Elmosallamy et al. developed an intelligent routing protocol that employs a Random Forest classifier for detailed Human Activity Recognition (HAR). The protocol dynamically adjusts its routing strategies, such as data transmission frequency and path priority, based on the classified activity—for instance, reducing transmission rates during resting to conserve energy and ensuring low-latency paths during exercising or emergencies. Table 4 summarizes these representative protocols. It is important to note that while these protocols all address challenges posed by human movement, they differ in their sensing mechanisms and response actions. EMA-RPL and ARMR focus on link-level metrics (RSSI trends and Link Lifetime Estimation), resulting in route switching actions on a micro-time scale. In contrast, MHRP and Elmosallamy et al. leverage macroscopic user-level context (posture and activity type). MHRP focuses on node activation to alter the physical topology, whereas Elmosallamy et al. prioritize adjusting transmission strategies (e.g., frequency). This diversity indicates a shift in movement-aware routing from simple link repair to context-aware system adaptation.

### 4.5. Cross-Layer Design Routing Protocol

The design philosophy of cross-layer routing protocols is to break the strict hierarchical constraints of traditional protocol stacks, enabling global collaborative optimization by allowing direct information exchange between non-adjacent layers. The key mechanism is allowing the routing layer to access physical or MAC layer information directly. An early example is the Priority-based Cross Layer Routing Protocol (PCLRP) [21] by Ben Elhadj et al. It utilizes MAC frames to construct the routing scheme and prioritizes traffic to meet QoS requirements, thus avoiding separate route discovery overhead. In [22], Zheng et al. proposed a protocol that dynamically adapts the MAC layer’s superframe structure and sleep/wake cycles across layers to address the dynamic channel and energy conditions in WBANs. Table 5 summarizes these representative protocols.

### 4.6. Fuzzy Logic-Based Routing Protocol

Fuzzy logic-based routing protocols utilize fuzzy logic systems to tackle decision-making problems that involve uncertainty. Their core technique is the fuzzification process, which converts specific, quantitative data (e.g., “remaining energy is 30%”) into vague, descriptive linguistic concepts. This transformation enables the protocol to make judgments that are, in essence, more akin to human reasoning and more robust when weighing conflicting objectives like energy consumption versus latency. Some research focuses on integrating fuzzy logic with multi-attribute decision-making (MADM) methods to introduce new decision-making dimensions. The FEELS [23] protocol incorporates biosafety metrics as a fuzzy input, leveraging fuzzy TOPSIS to achieve a robust balance between energy efficiency and safety. A more recent trend involves embedding fuzzy logic into complex hybrid intelligent frameworks. The SIMOF [24] protocol combines fuzzy logic with swarm intelligence algorithms to synergistically optimize stability periods and mitigate hotspot issues. The H-TERF [25] protocol integrates fuzzy multi-criteria decision-making with enhanced random forests to facilitate more precise routing decisions across multiple dimensions, including energy, delay, and throughput. These hybrid models exemplify a significant trend where fuzzy logic serves as a reasoning layer, harmonizing the outputs of various specialized algorithms for a more unified and intelligent decision-making process. Table 6 summarizes these representative protocols.

### 4.7. Software-Defined Networking Routing Protocol

The core idea of Software-Defined Networking (SDN) is to separate the network’s control functions from its data forwarding functions, enabling centralized management of routing decisions. In WBANs, a central controller maintains a global view of the network, computes optimal paths, and disseminates these paths to sensor nodes, which are responsible solely for forwarding. This centralized model simplifies network management and allows for consistent and global optimization of routing paths, making it well-suited for the dynamic WBAN environment. By offloading complex routing computations to the controller, it also reduces the processing burden on resource-constrained sensor nodes. The ESR-W [26] protocol employs a fuzzy logic-based Dijkstra’s Algorithm on the controller to compute optimal paths. The ERQTM [27] protocol further implements QoS assurance within the SDN framework. Table 7 summarizes these representative protocols.

### 4.8. Interference-Based Routing Protocol

The primary goal of interference-based routing protocols is to improve network reliability in complex wireless signal environments, with a focus on managing the mutual signal interference caused when multiple WBANs operate simultaneously. The ERS [28] protocol uses Latin squares for channel/slot assignment and a hash function for retransmissions. This facilitates conflict-free resource allocation without necessitating information exchange between nodes. The CORS [29] protocol further optimizes this approach by designing a more economical resource scheduling scheme through grouped Latin squares and differential hash functions, reducing interference while conserving network resources. Table 8 summarizes these representative protocols.

### 4.9. Machine Learning/Artificial Intelligence-Based Routing Protocol

Recent work has increasingly explored machine learning and optimization-based methods to make routing decisions using learned or computed policies. One category of these protocols uses traditional supervised learning models. Their mechanism involves training a predictive model on network feature data (such as a node’s remaining energy and inter-node distance). This model can then dynamically infer the optimal next-hop node, replacing static routing algorithms. Such as the approach by Benlaldj et al. [30], which uses decision trees and k-NN algorithms to classify and select the optimal next hop based on features like node energy and distance. Another prominent branch of research leverages bio-inspired optimization algorithms, which draw inspiration from natural phenomena to solve complex problems. Others focus on bio-inspired optimization algorithms; for instance, the BFO-LRP [31] protocol applies the drosophila optimization algorithm (BFO) to find routing paths that minimize packet delay. Expanding on this theme, the EEART protocol [32] employs Glowworm Swarm Optimization (GSO), where nodes mimic glowworms and use a luciferin value representing path fitness to find routes that optimize both energy and delay. In a similar vein, the DERP protocol [33] utilizes a Differential Evolution (DE) algorithm. This approach seeks an optimal path by evolving a population of potential routes against a fitness function that considers both energy consumption and link stability, thereby addressing node mobility. To provide a consistent analytical framework for comparing the optimization goals of these bio-inspired algorithms, we define a generalized fitness function template *F = f* (*E*, *D*, *S*), where *E*, *D*, and *S* represent energy efficiency, delay, and link stability, respectively. The protocols differ in their argument selection and optimization directions as follows: * BFO-LRP: Focuses on a single-objective function *F = f* (*D*), with the primary goal of minimizing *D* (packet delay). * EEART: Uses a luciferin value to represent fitness *F = f* (*E*, *D*), aiming to maximize *E* (energy efficiency) while simultaneously minimizing *D* (delay). * DERP: Employs a fitness function *F = f* (*E*, *S*), evolving routes to optimize the trade-off between minimizing energy consumption and maximizing *S* (link stability). The MDML-RP [34] protocol represents a more advanced strategy, supporting fast, real-time machine learning-based routing decisions during online operation through offline metaheuristic algorithm training. Additionally, another approach positions the routing protocol as a foundational support for higher-level deep learning applications (like disease detection). The RPESA [35] algorithm is designed for this purpose, with its main task being to provide energy-efficient data transmission services for these applications. Table 9 summarizes these representative protocols.

## 5. Comparative Analysis and Discussion

This section offers an overview and discussion of the aforementioned routing protocols, assesses the recent state of research through quantitative comparisons, examines the core principles of WBAN routing design, and discusses the design principles for WBAN routing protocols.

### 5.1. Analysis of Routing Control Scopes

Before presenting the statistical and performance analysis, it is crucial to synthesize the operational mechanisms of the protocols reviewed in Section 4. While these protocols utilize diverse algorithms ranging from swarm intelligence to fuzzy logic, their specific interventions in the routing process vary significantly. To provide a unified description, Table 10 maps each protocol category and its representative works to their primary Algorithm Control Scope. This summary clarifies whether an algorithm specifically governs topological management (e.g., cluster head selection), path decision-making (e.g., relay selection), or resource allocation (e.g., time-slot scheduling), thereby offering a view of the control logic inherent in modern WBAN routing.

### 5.2. Category Distribution

**Figure 2:**
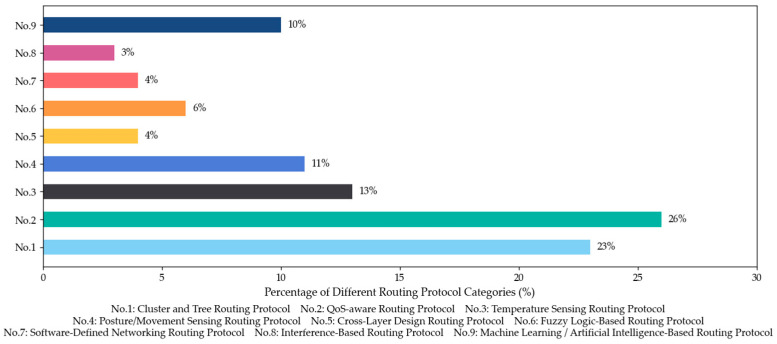
Distribution of WBAN routing protocol categories.

To visually illustrate the research landscape of WBAN routing protocols, we conducted a statistical analysis of academic literature, as shown in Figure 2. The data reveal that QoS-aware routing protocols account for the largest share at 26%, underscoring their critical importance for medical data transmission. Cluster and tree routing protocols follow closely at 23%, indicating their widespread adoption for enhancing energy efficiency. Key challenge-oriented approaches, including temperature-sensing, posture/movement-sensing, and machine learning/artificial intelligence-based protocols, represent the next significant tier of research, constituting 13%, 11%, and 10% respectively. The remaining categories are more specialized, with fuzzy logic-based protocols at 6%, while cross-layer design and software-defined networking protocols each account for 4%. Interference-based protocols comprise the smallest portion at 3%. This distribution provides a clear overview of the recent research landscape, highlighting the primary focus on QoS and energy efficiency, followed by a diverse range of context-aware and intelligent solutions.

Beyond the static distribution of categories, a temporal analysis of the selected literature reveals a distinct evolution in research focus. During the earlier phase of our review window (2020–2022), research primarily concentrated on addressing fundamental physical constraints, such as energy consumption and interference, through various clustering and tree-based structures. However, in the most recent years (2023–2025), there has been a trend toward intelligence and predictiveness. As reflected in the prevalence of QoS-aware and Machine Learning-based protocols in the latest publications, the field is moving beyond simple energy conservation toward more advanced, adaptive algorithms capable of handling complex, heterogeneous traffic and dynamic environmental contexts.

### 5.3. Performance Comparison

A fair comparison of WBAN routing protocols necessitates an evaluation across multiple performance dimensions, as the unique constraints of the on-body environment mean no single metric determines overall effectiveness. To compare these protocols fairly, we evaluate them on six core dimensions. Energy efficiency is paramount for maximizing the network’s operational lifespan, as minimizing consumption directly reduces the frequency of intrusive battery replacements for implanted or wearable nodes. Failing to optimize for energy could compromise the viability of long-term monitoring and even necessitate premature surgical procedures for device replacement. Low latency and reliability are crucial for clinical viability; low latency ensures the timely delivery of critical alerts, while reliability guarantees that vital health information is not lost in transit. Security is a fundamental requirement for protecting sensitive patient data, encompassing confidentiality against unauthorized access, integrity, and authenticity. A breach not only compromises patient privacy but could also allow for malicious data manipulation, posing a direct threat to patient health. We also assess computational complexity, regarding processing power and memory; given the severe resource constraints of WBAN nodes, high complexity can make implementation infeasible or drain batteries too quickly. To elaborate on the computational complexity assessments in Table 11, it is vital to consider the algorithmic time complexity relative to node density (*N*). Simple mechanisms, such as location-based greedy forwarding, typically operate with *O*(*N*) or even *O*(1) complexity per hop using local neighbor tables. In contrast, cluster-based protocols often incur *O*(*N*) overhead during the setup phase for head selection. The highest complexity arises in AI-based and Swarm Intelligence protocols (e.g., ACO, GWO), where the iterative optimization process can reach a time complexity of *O*(*T·P·N*), where *T* is the number of iterations and *P* is the population size. While these algorithms offer superior path quality, their implementation on resource-constrained 8-bit or 16-bit microcontrollers requires a careful trade-off between convergence precision and processing cycles. Lastly, scalability and mobility gauge robustness in dynamic environments, ensuring protocols perform efficiently as sensor numbers increase and remain resilient to topological changes caused by user movement. A protocol must therefore gracefully handle the frequent disconnections and reconnections that are characteristic of a body in motion. Table 11 summarizes this multi-faceted analysis, assigning qualitative ratings to each category to provide a holistic view of their strengths and weaknesses. We defined qualitative criteria based on the protocols’ intrinsic mechanisms. “High” energy efficiency denotes protocols utilizing mechanisms like sleep scheduling or hierarchical aggregation to significantly reduce idle listening. “High” reliability indicates the presence of multi-path transmission or retransmission schemes. “High” computational complexity refers to algorithms involving iterative optimization (e.g., Swarm Intelligence) or complex cryptographic operations, whereas “Low” complexity characterizes simple greedy forwarding or static routing.

The table illustrates the inherent performance trade-offs among different WBAN routing protocol categories. These ratings derive from an overview analysis of core design principles and literature consensus, rather than a single quantitative simulation. The analysis confirms a no free lunch scenario where no universally optimal protocol exists; improving one metric often necessitates sacrificing another due to fundamental interdependencies. For instance, mechanisms for high energy efficiency, such as sleep cycles, inherently increase latency. Conversely, ensuring reliability amidst movement requires frequent signaling, which consumes additional energy, while adding security encryption introduces computational overhead. Each category reflects a distinct design philosophy. Cluster and tree protocols are highly optimized for energy efficiency but can introduce latency due to their hierarchical structure. In contrast, posture/movement-sensing protocols excel in reliability and mobility support by adapting to the dynamic environment, though at the cost of higher computational complexity. Other specialized protocols, such as QoS-aware ones, are tailored for specific objectives, reinforcing that every design choice involves compromise. Ultimately, protocol selection is about finding the best-fit solution for a specific application’s unique requirements.

### 5.4. Design Principles for WBAN Routing Protocols

Through the analysis and discussion of the various protocols mentioned above, we have identified several core principles that should be followed in designing the next-generation WBAN routing protocol:

1. Application-Driven Design: Protocol design should begin with a deep understanding of specific application requirements. For instance, critical monitoring demands the lowest latency, whereas routine monitoring prioritizes maximum lifespan. Defining the primary optimization goal is the first step in making performance characteristics.
2. Multi-Objective Optimization: Moving beyond single-metric optimization, this approach employs weighted cost functions, fuzzy logic, and multi-objective optimization algorithms to evaluate and synergistically optimize multiple dimensions, including energy efficiency, latency, reliability, security, and computational complexity. To rigorously define this process, the optimization goal is typically formulated as minimizing a composite cost function *J*:
  (1) $$J=\sum_{i=1}^{K}w_{i}\cdot m_{i}$$
  where *K* represents the total number of optimization objectives. Each $m_{i}$ denotes a normalized performance metric derived from quantities such as energy consumption (E), end-to-end latency (L), link unreliability (1 − R), node temperature (T), or security risk (S). To ensure the problem is well-posed and reflects application-specific preferences, the weighting coefficients $w_{i}$ must satisfy the constraints:
  (2) $$w_{i}\geq 0,\;\sum_{i=1}^{K}w_{i}=1$$
  This mathematical formulation allows the protocol to adapt to varying QoS requirements by dynamically adjusting $w_{i}$ (e.g., significantly increasing the weight of the latency metric in emergency modes).

3. Adaptability: The WBAN environment is highly dynamic. Protocols must possess the ability to sense and adapt to environmental changes, whether shifts in user posture, fluctuations in channel quality, or variations in network traffic load. Introducing predictive mechanisms and learning capabilities (such as AI/ML) is key to achieving advanced adaptability.
4. Lightweight Design: Given the resource constraints of nodes, all protocol mechanisms—whether routing computation, signaling exchange, or security encryption—must pursue extreme lightweight design. This ensures the required functionality is achieved with minimal computational and communication overhead.

### 5.5. Critical Analysis: Practicality and Security

Beyond performance metrics, assessing the practical deployment potential and security of protocols is critical.

1. Theoretical Concepts vs. Practical Deployment: Currently, some cluster and tree-based routing protocols are relatively straightforward to implement and are compatible with common low-power radios, so they may be easier to prototype than designs that require heavy online learning or centralized control. However, practical feasibility still depends on hardware, synchronization, and mobility conditions. In contrast, many AI-based and SDN-based proposals are primarily evaluated in simulations, and their real-world cost (computation, control overhead, robustness) needs further validation. While they demonstrate superior performance in simulations, their high demands for computational resources and centralized control often exceed the processing capabilities of current commercial miniature sensors, hindering near-term large-scale implementation.
2. Security and Privacy: This is a critical but often overlooked area. Most reviewed protocols focus solely on routing efficiency, neglecting security. Since WBANs handle sensitive health data, protocols must be resilient against attacks (e.g., blackhole, selective forwarding). However, implementing robust encryption (e.g., AES) and key management mechanisms significantly increases energy consumption. Future designs must strike a balance between routing efficiency and lightweight security mechanisms; otherwise, “efficient” routing will remain unacceptable for clinical applications.

## 6. Open Challenges and Future Directions

Following the analysis of WBAN routing protocols in previous sections, this section will discuss the core routing challenges that remain unresolved in WBAN routing protocols and explore the integration of cutting-edge technologies.

### 6.1. Unresolved Routing Challenges

Despite significant progress across various dimensions in existing research, designing a routing protocol capable of long-term stable operation in real, complex, and dynamic human environments still faces numerous unresolved core challenges.

1. Multi-Objective Optimization: While modern designs favor multi-objective optimization, most still rely on static linear weighting with empirical coefficients, which limits adaptability across different scenarios. A critical theoretical gap remains in establishing an adaptive framework that can dynamically adjust the weights of conflicting performance metrics. The challenge lies in solving this complex optimization problem efficiently on resource-constrained nodes without relying on fixed, predefined parameters.
2. Robustness in Dynamic Environments: recent posture/movement-sensing routing protocols effectively handle predictable motions but often fail during the violent, unpredictable topology changes characteristic of extreme scenarios, such as high-intensity sports or disaster rescue operations. Designing a highly robust protocol that maintains millisecond-level latency and near-perfect reliability under these extreme dynamic conditions remains a significant technical hurdle.
3. Routing for Energy Harvesting Networks: Energy harvesting offers a sustainable power source but introduces instability due to the intermittent and unpredictable nature of energy acquisition. Future protocols must shift to an opportunistic paradigm that not only detects recent energy levels but also predicts future harvesting rates. The key challenge is to intelligently direct data flows to nodes with sufficient or replenishing energy, ensuring continuous network operation and maximizing throughput. This transforms the routing decision from a simple cost calculation into a complex optimization problem, balancing immediate transmission needs with long-term network sustainability.

### 6.2. Integration with Emerging Technologies

The development of emerging technologies, particularly artificial intelligence, edge computing, and next-generation communication technologies, offers opportunities to overcome the aforementioned challenges and reshape WBAN routing protocols.

1. Artificial Intelligence: AI/ML-based routing protocols have begun to emerge, with future integration set to deepen significantly. Online Reinforcement Learning may allow nodes to achieve real-time, adaptive self-learning of routing strategies without offline training. Federated Learning allows multiple WBANs to collaboratively train robust models in a privacy-preserving manner, which is crucial for large-scale medical IoT systems.
2. Edge and Fog Computing: Offloading complex routing computations and AI inference to powerful edge devices effectively addresses on-node computational bottlenecks. This approach allows WBANs to run more sophisticated, centralized optimization algorithms—like deep reinforcement learning—by combining SDN principles with edge computing, thus improving system-level performance without burdening the sensor nodes.
3. 6G Networks and ISAC: Future wireless systems (including beyond-5G/6G concepts) may help improve latency and reliability for medical IoT scenarios, although their applicability to on-body networks will depend on device size, power, and regulation. Integrated sensing and communications (ISAC) could potentially provide additional context information (e.g., motion-related channel features) that may assist link prediction, but the feasibility and energy cost for WBAN nodes still require further study.
4. Blockchain and Digital Twins: In multi-WBAN scenarios, blockchain can provide a secure, decentralized platform for managing routing decisions and relay selection. Meanwhile, digital twin technology uses this reliably routed data to build a real-time virtual model of the user for predictive simulations. The outcomes are then fed back to the WBAN, creating an intelligent closed-loop system that dynamically optimizes monitoring and routing priorities.

## 7. Conclusions

This paper reviewed representative work on WBAN routing protocols and addressed the three research questions stated in the introduction. For RQ1, we proposed a taxonomy with nine categories; within the papers covered in this review, QoS-aware and cluster-based protocols appear frequently. For RQ2, we compared protocols across key performance aspects and noted common trade-offs among metrics such as energy efficiency and latency, suggesting that a single protocol is unlikely to be optimal for all scenarios. For RQ3, we summarized open issues, including adaptive multi-objective weighted optimization and robustness under highly dynamic conditions, and discussed AI, edge computing, and 6G-related technologies as potential future research directions.

Looking ahead, emerging technologies like AI, Edge/Fog computing, and 6G networks will guide the future of WBAN routing and offer solutions to recent limitations. In conclusion, this survey provides an overview of the field. By classifying and comparing existing approaches, it serves as a reference for researchers and identifies key areas for future work.

## Figures and Tables

**Table 1:** Cluster and Tree Routing Protocols.

Protocol Name	Key Challenges Addressed	Limitations
MGWOQL	Minimizing energy consumption by using machine learning to predict energy wastage and a hybrid GWO with Q-learning for optimal CH selection.	The integration of Q-learning adds another layer of computational complexity and requires careful tuning of learning parameters.
ABC-CSO	Conserving energy and extending network lifespan through hierarchical data aggregation.	Increased computational complexity due to the hybrid swarm intelligence algorithm.
ABBSOA-MSSOA	Improving energy efficiency and balancing network load via optimized cluster formation and routing.	High computational complexity from combining two distinct swarm optimization algorithms; potential for increased latency due to hierarchical structure.

**Table 2:** QoS-aware Routing Protocols.

Protocol Name	Key Challenges Addressed	Limitations
PCRP, EQRD, IM-QRP	Prioritizing critical data to minimize latency.	Increased energy consumption for low-latency paths.
Tripe-EEC	Granular prioritization via multi-level data classification.	Complex management of multiple data classes.
ARAP	Balancing conflicting QoS metrics for optimal path selection.	High computational overhead from MCDM/TOPSIS algorithms.

**Table 3:** Temperature-Sensing Routing Protocols.

Protocol Name	Key Challenges Addressed	Limitations
ATAR	Preventing tissue overheating by finding alternative routes when hotspots are detected.	Its reactive nature might introduce a delay in finding a new path after a hotspot has already formed.
EOCC-TARA	Co-optimizing temperature, energy consumption, and network congestion in a centralized, SDN-based manner.	Relies on an SDN framework, which can be a single point of failure; the EMSMO algorithm is computationally complex.
SETA	Preventing tissue overheating from RF radiation while maintaining energy efficiency and link quality.	The enhanced Ant Colony Optimization (ACO) algorithm can be computationally intensive.
ETC-MOTLBO	Co-optimizing temperature, energy consumption, and network congestion in a centralized manner.	Relies on an SDN framework, which can be a single point of failure and introduces control overhead.
TSA-ESRP	Dynamically adjust routing by node thermal state to avoid hotspots.	Discretized temp states offer less granular control vs. continuous monitoring.

**Table 4:** Posture/Movement-Sensing Routing Protocols.

Protocol Name	Key Challenges Addressed	Limitations
EMA-RPL	Proactively avoiding link failures caused by user movement by predicting link degradation.	Proactive switching is based on RSSI prediction, which can sometimes be unreliable in complex channel environments.
ARMR	Intelligently learning and selecting more reliable and durable routes in dynamic topologies.	Reinforcement learning introduces learning overhead and complexity, and may be slow to adapt to sudden, drastic movements.
MHRP	Maintaining stable communication paths by activating posture-specific nodes.	Requires pre-deployment of redundant nodes, which increases the initial hardware cost and network density.
Elmosallamy et al.	Adapting routing strategies (e.g., data rate) based on fine-grained recognition of user activities (resting, walking, etc.).	Relies on a machine learning model (Random Forest), which adds computational complexity for activity classification.

**Table 5:** Cross-Layer Design Routing Protocols.

Protocol Name	Key Challenges Addressed	Limitations
PCLRP	Providing differentiated QoS by using MAC layer information to guide routing decisions without explicit route discovery.	Performance depends on a specific custom MAC, which reduces modularity and may not achieve full global optimization.
Zheng et al.	Globally optimizing energy and channel adaptation by breaking the rigid protocol stack hierarchy.	Leads to reduced protocol modularity and increased design complexity, making maintenance and updates more difficult.

**Table 6:** Fuzzy Logic-Based Routing Protocols.

Protocol Name	Key Challenges Addressed	Limitations
FEELS	Making robust routing decisions under uncertainty, incorporating biosafety (SAR) as a decision metric.	The process of fuzzification, inference, and defuzzification adds computational overhead.
SIMOF	Balancing multiple conflicting objectives (e.g., stability, hotspots) by combining fuzzy logic with swarm intelligence algorithms.	The hybrid framework of fuzzy logic and swarm intelligence significantly increases computational complexity.
H-TERF	Integrating fuzzy logic and random forests for precise, multi-objective routing decisions.	High computational complexity due to the complex hybrid framework.

**Table 7:** Software-Defined Networking Routing Protocols.

Protocol Name	Key Challenges Addressed	Limitations
ESR-W	Achieving globally optimal paths through centralized computation using fuzzy logic-based algorithms on the controller.	The central controller can become a performance bottleneck or a single point of failure.
ERQTM	Implementing centralized QoS management and traffic control to ensure differentiated services within the SDN framework.	The centralized architecture introduces control overhead from constant node-controller communication and represents a potential single point of failure.

**Table 8:** Interference-Based Routing Protocols.

Protocol Name	Key Challenges Addressed	Limitations
ERS	Mitigating interference in co-located WBANs through conflict-free channel/slot assignment using Latin squares.	The resource allocation scheme might not be the most economical, potentially reserving more resources than necessary, leading to underutilization.
CORS	More resource-efficient interference mitigation.	The design of grouped Latin squares and differential hash functions adds significant algorithmic complexity.

**Table 9:** Machine Learning/Artificial Intelligence-Based Routing Protocols.

Protocol Name	Key Challenges Addressed	Limitations
Benlaldj et al.	Predicting optimal next-hop via learned features to save energy.	Requires labeled datasets; sensitive to training feature quality.
BFO-LRP, EEART, DERP	Finding near-optimal paths for energy, delay, and stability.	High computational cost; unpredictable convergence time.
MDML-RP	Enabling fast, real-time routing adaptation to dynamic network conditions based on offline training.	The effectiveness relies on the quality of the offline metaheuristic training phase and may not generalize well to unforeseen network scenarios.
RPESA	Energy-efficient routing designed to support a specific on-body deep learning application.	Highly application-specific, which limits its general-purpose usability.

**Table 10:** Mapping of Algorithms to Routing Control Scopes.

Protocol Category	Representative Protocols	Primary Algorithm Control Scope
Cluster and Tree Routing	MGWOQL, ABC-CSO, ABBSOA-MSSOA	Topology Management: Controls Cluster Head (CH) election and cluster formation.
QoS-aware Routing	PCRP, EQRD, Tripe-EEC, ARAP	Path Selection: Controls packet prioritization and next-hop relay selection.
Temperature-Sensing Routing	ATAR, EOCC-TARA, SETA, TSA-ESRP	Route Maintenance: Controls path redirection and traffic distribution to avoid hotspots.
Posture/Movement-Sensing Routing	EMA-RPL, ARMR, MHRP	Link Prediction: Controls proactive route switching and node activation.
Cross-Layer Design Routing	PCLRP, Zheng et al.	Resource Allocation: Jointly controls MAC time-slots and forwarding paths.
Fuzzy Logic-Based Routing	FEELS, SIMOF, H-TERF	Decision-Making: Controls multi-criteria weighting for next-hop selection.
Software-Defined Networking (SDN)	ESR-W, ERQTM	Global Computation: Controls centralized path calculation and flow rule generation.
Interference-Based Routing	ERS, CORS	Channel Access: Controls frequency assignment and time-slot scheduling.
Machine Learning/AI-Based Routing	BFO-LRP, EEART, DERP, Benlaldj et al.	Path Fitness Evaluation: Controls next-hop prediction and route evolution by learning from network features (energy, delay, etc.).

**Table 11:** Performance Comparison of WBAN Routing Protocol Categories.

Protocol Category	Energy Efficiency	Low Latency	Reliability	Security	Computational Complexity	Scalability/Mobility
Cluster and Tree Routing	High	Low	Medium	Medium	Medium	Low
QoS-aware Routing	Low	High	High	Medium	Medium	Medium
Temperature-Sensing Routing	High	Medium	Medium	Low	Medium	Medium
Posture/Movement Sensing	Medium	Medium	High	Medium	High	High
Cross-Layer Design Routing	Medium	High	High	Low	High	Medium
Fuzzy Logic-Based Routing	Medium	Medium	High	Low	High	Medium
Software-Defined Networking	Medium	High	High	High	Low (on nodes)	High
Interference-Based Routing	Medium	Medium	Medium	Low	High	Low
Machine Learning/AI-Based	High	Medium	High	Medium	High	High

## Data Availability

Data are contained within the article.

## Abbreviations

The following abbreviations are used in this manuscript:

WBANs	Wireless Body Area Networks
WSNs	Wireless Sensor Networks
QoS	Quality of Service
CH	Cluster Head
SDN	Software-Defined Network
RL	Reinforcement Learning
LLE	Link Lifetime Estimation
PD	Personal Device
EHR	Electronic Health Records
RSSI	Received Signal Strength Indicator
HAR	Human Activity Recognition
BLE	Bluetooth Low Energy

## References

[1] Narwal B., Malik M., Mohapatra A.K., Baliyan N., Shukla V., Kumar M. (2024). Dissecting wireless body area networks routing protocols: Classification, comparative analysis, and research challenges. Int. J. Commun. Syst..

[2] Qin H., Su H., Niu X., Chen H. The State-of-the-Art Routing Protocols for Wireless Body Area Networks: A Survey. Proceedings of the 2025 10th International Conference on Computer and Information Processing Technology (ISCIPT).

[3] Bedi P., Das S., Goyal S., Shukla P.K., Mirjalili S., Kumar M. (2022). A novel routing protocol based on grey wolf optimization and Q learning for wireless body area network. Expert Syst. Appl..

[4] Dinesh A., Rangaraj J. (2025). An energy-efficient routing protocol for wireless body area networks using hybrid artificial bee colony optimization and chicken swarm optimization algorithm. J. Eng. Appl. Sci..

[5] Sureshkumar S., Babu A.V.S., James S.J. (2025). Empowering WBANs: Enhanced energy efficiency through cluster-based routing and swarm optimization. Symmetry.

[6] Awan K.M., Ashraf N., Saleem M.Q., Sheta O.E., Qureshi K.N., Zeb A., Haseeb K., Sadiq A.S. (2019). A priority-based congestion-avoidance routing protocol using IoT-based heterogeneous medical sensors for energy efficiency in healthcare wireless body area networks. Int. J. Distrib. Sens. Netw..

[7] Kaur N., Verma S., Kavita, Jhanjhi N.Z., Singh S., Ghoniem R.M., Ray S.K. (2023). Enhanced QoS-aware routing protocol for delay sensitive data in wireless body area networks. IEEE Access.

[8] Ahmad N., Awan M.D., Khiyal M.S.H., Babar M.I., Abdelmaboud A., Ibrahim H.A., Hamed N.O. (2022). Improved QoS aware routing protocol (IM-QRP) for WBAN based healthcare monitoring system. IEEE Access.

[9] Ullah F., Ullah Z., Ahmad S., Islam I.U., Rehman S.U., Iqbal J. (2019). Traffic priority based delay-aware and energy efficient path allocation routing protocol for wireless body area network. J. Ambient Intell. Humaniz. Comput..

[10] Singh S., Bilandi N. (2025). Adaptive relay-assisted WBAN protocol: Enhancing energy efficiency and QoS through advanced multi-criteria decision-making. Comput. Model. Eng. Sci..

[11] Jamil F., Iqbal M.A., Amin R., Kim D. (2019). Adaptive thermal-aware routing protocol for wireless body area network. Electronics.

[12] Ahmed O., Ren F., Hawbani A., Al-Sharabi Y. (2020). Energy optimized congestion control-based temperature aware routing algorithm for software defined wireless body area networks. IEEE Access.

[13] Abdullah A.M. (2025). Energy-efficient and thermal-aware routing protocol for wireless body area networks based on ant colony optimization. Computing.

[14] Mozaffari J., Azgomi M.A., Madadi H., Dishabi M.R.E. (2025). Energy and temperature-aware routing approach for congestion control in wireless body area networks. J. Supercomput..

[15] Mu Y., Zheng G., Wang X., Zhu M., Ma H. (2025). Temperature state awareness-based energy-saving routing protocol for wireless body area network. Appl. Sci..

[16] Bouaziz M., Rachedi A., Belghith A. (2019). EMA-RPL: Energy and mobility aware routing for the Internet of Mobile Things. Future Gener. Comput. Syst..

[17] Ehsanfar S., Bazzi A., Mößner K., Chafii M. Hypothesis Testing on FMCW and OFDM for Joint Communication and Radar in IEEE 802.11bd. Proceedings of the 2023 IEEE International Conference on Communications Workshops (ICC Workshops).

[18] Arafat M.Y., Pan S., Bak E. (2024). An adaptive reinforcement learning-based mobility-aware routing for heterogeneous wireless body area networks. IEEE Sens. J..

[19] Karmakar K., Biswas S., Neogy S. MHRP: A novel mobility handling routing protocol in wireless body area network. Proceedings of the IEEE International Conference on Wireless Communications, Signal Processing and Networking (WiSPNET).

[20] Elmosallamy E.S., Soliman M.F. (2025). Intelligent routing for human activity recognition in wireless body area networks. Sci. Rep..

[21] Ben Elhadj H., Elias J., Chaari L., Kamoun L. (2016). A priority based cross layer routing protocol for healthcare applications. Ad Hoc Netw..

[22] Zheng L., Hu J., Jiao Y. (2023). A cross-layer media access control protocol for WBANs. Sustainability.

[23] Zadoo M., Sharma M., Choudhary A. (2022). FEELS: Fuzzy based energy efficient and low SAR routing protocol for wireless body area networks. Wirel. Netw..

[24] Aryai P., Khademzadeh A., Jassbi S.J., Hosseinzadeh M. (2023). SIMOF: Swarm intelligence multi-objective fuzzy thermal-aware routing protocol for WBANs. J. Supercomput..

[25] Khoshvaght P., Tanveer J., Rahmani A.M., Mohammadi M., Mehranzadeh A., Lansky J., Hosseinzadeh M. (2025). H-TERF: A hybrid approach combining fuzzy multi-criteria decision-making techniques and enhanced random forest to improve WBAN-IoT. Internet Things.

[26] Cicioğlu M., Çalhan A. (2020). Energy-efficient and SDN-enabled routing algorithm for wireless body area networks. Comput. Commun..

[27] Samarji N., Salamah M. (2021). ERQTM: Energy-efficient routing and QoS-supported traffic management scheme for SDWBANs. IEEE Sens. J..

[28] Fan L., Liu X., Zhou H., Leung V.C.M., Su J., Liu A.X. (2023). Efficient resource scheduling for interference alleviation in dynamic coexisting WBANs. IEEE Trans. Mob. Comput..

[29] Huang S., Liu X., Zhou H., Leung V.C.M. (2025). Conservation-oriented resource scheduling for interference mitigation in dynamic coexisting wireless body area networks. IEEE Trans. Green Commun. Netw..

[30] Benlaldj L., Hachemi M.H., Mhamedi M., Hadjila M., Bekkouche A. (2025). Machine learning-based energy-aware routing for wireless body area networks. Eng. Technol. Appl. Sci. Res..

[31] Veerathinaku S., Devanathan B. (2025). Breakthrough fruitfly optimization-based leach routing protocol (BFO-LRP) for packet delay minimization in wireless body area networking (WBAN). J. Theor. Appl. Inf. Technol..

[32] Goel S., Guleria K., Panda S.N., Alharithi F.S., Singh A., Ali A. (2025). An improved routing technique for energy optimization and delay reduction for wireless body area networks. Egypt. Inform. J..

[33] Smail O., Soltani K., Mekkaoui A. (2025). DERP: Differential evolution based routing protocol for wireless body area networks. J. Inf. Sci. Eng..

[34] Aryai P., Khademzadeh A., Jassbi S.J., Hosseinzadeh M., Hashemzadeh O., Shokouhifar M. (2023). Real-time health monitoring in WBANs using hybrid metaheuristic-driven machine learning routing protocol (MDML-RP). Int. J. Electron. Commun. (AEU).

[35] Liya B.S., Krishnamoorthy R., Arun S. (2024). An enhanced deep learning-based disease detection model in wireless body area network with energy efficient routing protocol. Wirel. Netw..

